# Comparing the effects of left bundle branch pacing and leadless right ventricular pacing on intraventricular and interventricular dyssynchrony using *in silico* modelling

**DOI:** 10.3389/fphys.2025.1644520

**Published:** 2025-10-08

**Authors:** Alphonsus Liew, Marina Strocchi, Cristobal Rodero, Karli K. Gillette, Nadeev Wijesuriya, Sandra Howell, Felicity de Vere, Edward J. Vigmond, Gernot Plank, Steven Niederer, Christopher Aldo Rinaldi

**Affiliations:** ^1^ School of Biomedical Engineering and Imaging Sciences, King’s College London, London, United Kingdom; ^2^ Department of Cardiology, Guy’s and St Thomas’ NHS Foundation Trust, London, United Kingdom; ^3^ National Heart and Lung Institute, Imperial College London, London, United Kingdom; ^4^ Medical University of Graz, Graz, Austria; ^5^ Department of Biomedical Engineering, University of Utah, Salt Lake City, UT, United States; ^6^ Scientific Computing and Imaging Institute, University of Utah, Salt Lake City, UT, United States; ^7^ University of Bordeaux, Centre National de la Recherche Scientifique, Bordeaux, France; ^8^ IHU Liryc, Bordeaux, France; ^9^ BioTechMed-Graz, Graz, Austria; ^10^ The Alan Turing Institute, London, United Kingdom

**Keywords:** left bundle branch pacing, leadless right ventricular pacing, intraventricular dyssynchrony, interventricular dyssynchrony, *in silico* modelling

## Abstract

**Introduction:**

Non-physiological right ventricular pacing (RVP) is currently the mainstay of treatment for patients with high-degree atrioventricular (AV) block who have preserved left ventricular ejection fraction. Newer pacing strategies, such as left bundle branch pacing (LBBP) and leadless cardiac pacemakers (LCPMs), are increasingly being adopted due to their respective advantages over RVP. However, there has been no direct comparison between LCPMs and LBBP regarding their risk of pacing-induced cardiomyopathy, which is thought to arise from interventricular and intraventricular dyssynchrony. Using *in silico* modelling, we compared the effects of LBBP and LCPMs on interventricular and intraventricular synchrony.

**Methods:**

Using 19 four-chamber healthy heart geometries, we simulated LCPMs at the level of the right ventricular outflow tract-septum (RVOT-S), mid-septum (MS), and apical septum (AS), along with proximal left bundle pacing (PLBBP) and distal left bundle pacing (DLBBP) in 3 different settings: 1) intact left bundle branch conduction, 2) left bundle branch block (LBBB), and 3) septal scar involving the His-Purkinje system (HPS). Ventricular electrical uncoupling (VEU), absolute VEU, and left ventricular dyssynchrony index (LVDI) were measured. The shortest interval required to activate 90% of both ventricles (BIVAT-90) was also recorded.

**Results:**

In the setting of intact left bundle branch conduction, combined LBBP configurations had significantly lower VEU (LBBP: −3.3 ± 5.1 vs. LCPM: 24.2 ± 7.6 ms, *p* < 0.01) and absolute VEU (LBBP: 5.0 ± 3.5 vs. LCPM: 24.2 ± 7.6 ms, *p* < 0.01) than combined LCPM configurations. In the presence of proximal LBBB, combined LBBP configurations also had significantly lower VEU (LBBP −22.1 ± 0.5 vs. LCPM 25.9 ± 7.9, *p* < 0.01) and absolute VEU (LBBP 22.1 ± 0.5 vs. LCPM 25.9 ± 7.9 ms, *p* < 0.01) than combined LCPM configurations. However, there was no significant difference in absolute VEU when combined LBBP configurations was compared with RVOT-S configuration alone (LBBP 22.1 ± 0.5 vs. RVOT-S 21.7 ± 9.0 ms, *p* = 0.86). In the presence of septal scar, combined LCPM configurations had significantly lower VEU compared with combined LBBP configurations (VEU: LCPM 31.0 ± 8.4 vs. LBBP 41.7 ± 20.2 ms, respectively; *p* < 0.01). Combined LBBP configurations had significantly lower LVDI and BIVAT-90 compared with combined LCPM configurations in both the presence and absence of LBBB, but there was no significant difference between the two in the setting of a septal scar.

**Conclusion:**

LCPM produces less interventricular dyssynchrony than LBBP in the presence of extensive septal scarring involving the HPS. In the setting of proximal LBBB, LCPM at the RVOT-S level may be non-inferior to LBBP in terms of interventricular dyssynchrony.

## 1 Introduction

Non-physiological right ventricular pacing (RVP) is currently the mainstay treatment for high-degree atrioventricular (AV) block. However, RVP is associated with the increased risk of pacing-induced cardiomyopathy, tricuspid regurgitation progression, and right ventricular dysfunction (Höke et al., 2014; Kanawati et al., 2021; Riesenhuber et al., 2021; Tatum et al., 2021; Chung et al., 2023; Boyle et al., 2024). In recent years, alternative forms of ventricular pacing, including left bundle branch pacing (LBBP) and leadless right ventricular pacing (LCPM), have been increasingly adopted due to their respective advantages over RVP. LBBP provides more physiological activation of the ventricles by engaging the His-Purkinje system (HPS), whereas LCPMs have been associated with significantly reduced progression of tricuspid regurgitation compared with RVP (Salaun et al., 2018; Garweg et al., 2023; El-Chami et al., 2024).

Both European and American guidelines recommend the use of cardiac resynchronisation therapy (CRT) in patients with an indication for ventricular pacing and impaired LV ejection fraction (LVEF) (Glikson et al., 2021; Chung et al., 2023). The European guidelines use a lower LVEF cut-off of <40% (Class I, Level A recommendation), whereas the American guidelines use a cut-off of <50% (2a, B-NR recommendation). However, in patients with preserved LVEF (i.e., >50%), the benefit of CRT is less clear, even in those with anticipated high ventricular pacing burden (Funck et al., 2025). LBBP prevents pacing-induced cardiomyopathy in those with preserved LVEF at baseline and preserves right ventricular function, but current available evidence points towards an increased risk of tricuspid regurgitation progression with LBBP (Chung et al., 2023; Hu et al., 2023; Li et al., 2023; Tian et al., 2023; Bednarek et al., 2024). In contrast, studies, including the 5-year Micra registry and a meta-analysis, have found that LCPM is associated with a significantly lower risk of tricuspid regurgitation (TR) progression (Salaun et al., 2018; Garweg et al., 2023; El-Chami et al., 2024; Yuyun et al., 2024). It is important to note, however, that septal positioning of LCPMs may exacerbate TR due to interaction with the tricuspid valve (Beurskens et al., 2019; Hai et al., 2021). Furthermore, LCPM eliminates the risk of pocket- and lead-related issues, including infection, lead fracture, pneumothorax, and haematoma (Salaun et al., 2018; Garweg et al., 2023). Moreover, LCPM appears to have significantly lower rates of pacing-induced cardiomyopathy when placed in a high septal position compared to RVP (0.3%–4% vs. 10%–25%, respectively). This makes LCPM an attractive alternative in patients with AV block requiring pacing and preserved LVEF. However, the comparison between LCPM and LBBP in terms of pacing-induced dyssynchrony, which may lead to pacing-induced cardiomyopathy (Tops et al., 2007; Pastore et al., 2008; Fang et al., 2016; Bansal et al., 2019), remains poorly understood.

### 1.1 Role and clinical relevance of *in silico* modelling

The role of *in silico* modelling has expanded rapidly over the past few decades. In silico modelling allows hypotheses to be tested noninvasively in the first instance, thereby informing and providing justification for *in vivo* studies and maximising the likelihood of detecting relevant outcomes. Patients with a pacing indication exhibit heterogeneous responses to pacing, influenced by individual and anatomical differences. In addition, septal scarring affects the feasibility and efficacy of LBBP, but the extent of septal scarring is highly variable among individuals. In silico modelling enables direct comparison of LBBP and LCPM under identical conditions, such as in the presence of extensive septal scarring and proximal LBBB, ensuring that any observed differences can be solely attributable to the pacing strategy.

In this study, we aim to compare the effects of LBBP and LCPM on interventricular and intraventricular dyssynchrony in the setting of complete AV block and preserved LVEF using *in silico* modelling.

## 2 Methods

### 2.1 *In silico* modelling

To perform our electrophysiology simulations, we used 19 publicly available four-chamber heart geometries from healthy subjects, obtained from a previous study (Rodero et al., 2021). The heart meshes were composed of linear tetrahedral elements with an average resolution of approximately 1 mm. A His-Purkinje network was added to each heart geometry based on previous studies (Gillette et al., 2021; 2022; Pathmanathan et al., 2024), as described in the Supplementary Material. The His-Purkinje network included three LV (anterior, septal, and posterior) and two RV (septal and moderator band) fascicles that were used to initiate the activation of the ventricular myocardium during sinus rhythm. Ventricular activation was computed using a reaction–Eikonal model with the Cardiac Arrhythmia Research Package (CARP) (Vigmond et al., 2002; Neic et al., 2017). The His-Purkinje network was assigned a conduction velocity (CV) of 3 m/s, while the ventricular myocardium was modelled as a transversely isotropic conduction medium, with a CV of 0.6 m/s along the fibres and 0.24 m/s in the transverse direction, in accordance with normal CV ranges measured in mammals (Draper and Mya-Tu, 1959). Proximal LBBB was simulated by cutting the connection of the left bundle to the LV His-Purkinje system along the His. In the Supplementary Material, we outline the fascicles for each of the patient-specific geometries. We also demonstrate that the sinus rhythm activation simulated by the model is physiological, aligns with the Durrer maps (Durrer et al., 1970) and that the resulting activation metrics are within the ranges reported in the literature. Finally, we demonstrate that the simulated activation during LBBB leads to prolonged activation times and delayed LV activation, in line with data from the published literature (Neic et al., 2017).

### 2.2 Pacing simulations

**FIGURE 1:**
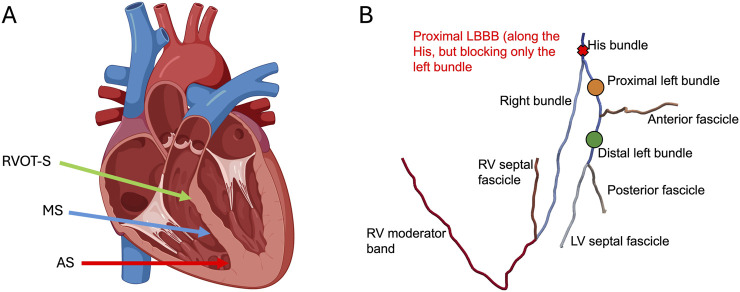
**(A)** Leadless cardiac pacing (LCPM) locations: right ventricular outflow tract-septum (RVOT-S) denoted by the green arrow, mid-septum (MS) denoted by the blue arrow, and apical septum (AS) denoted by the red arrow. **(B)** Simulated pacing locations of LBBP and proximal LBBB. Left bundle branch pacing was performed along the proximal left bundle (orange dot) and distal left bundle (green dot). Where present, LBBB was simulated at the level of the His, affecting only the left bundle (red cross).

Using the *in silico* model, we simulated ventricular activation during sinus rhythm under the conditions of intranodal block with junctional escape rhythm (AV node blocked but intact His bundle), LBBP in two configurations (Figure 1B)—proximal left bundle pacing (PLBBP) and distal left bundle pacing (DLBBP)—and LCPM in three configurations (Figure 1A): at the level of the RVOT-septum (RVOT-S), mid-septum (MS), and apical septum (AS). We considered three different baseline rhythm scenarios: 1) intact left bundle branch conduction, 2) left bundle branch block (LBBB) and 3) septal scar involving the HPS. Complete AV block at the level of the AV node was simulated in all pacing settings, such that the intrinsic rhythm did not contribute to ventricular activation.

### 2.3 Septal scar mapping

To simulate septal scarring, we integrated a patient-specific scar and border zone geometry from a publicly available 1 mm resolution LV patient-specific mesh into our 19 heart geometries using the universal ventricular coordinates (UVCs) (Bayer et al., 2018; Mendonca Costa et al., 2019). The septal scar was mapped to the meshes in our cohort (as shown in the Supplementary Material), and the scar core was simulated as non-conducting tissue. The Purkinje fibres overlapping the scar tissue were identified and assumed to be non-conducting, under the assumption that scarred Purkinje is affected by tissue hypoxia the same way as normal myocardium (Mendonca Costa et al., 2019).

### 2.4 Measures of intraventricular and interventricular dyssynchrony

To assess interventricular dyssynchrony, we computed ventricular electrical uncoupling (VEU) and absolute VEU. VEU was calculated as the difference between the mean LV and RV epicardial activation times (i.e., mean LV epicardial activation time–mean RV epicardial activation time) and indicates the directionality of dyssynchrony (a positive value indicates that LV takes longer to activate than RV, and a negative value indicates that RV takes longer to activate than LV). Absolute VEU values were also defined to reflect the degree of dyssynchrony between the LV and RV, irrespective of directionality. Left ventricular dyssynchrony index (LVDI) was used to represent LV intraventricular dyssynchrony and calculated as the standard deviation of activation times within the LV. The shortest interval taken to activate 90% of both ventricles (BIVAT-90) was used to reflect biventricular activation time. When computing response to pacing, the areas around the AV valves and outflow tracts were excluded.

### 2.5 Statistical analysis

Means and standard deviations were used to summarise and present continuous variables. The Shapiro–Wilk test was used to test for normality of continuous data. Two-tailed Student’s t-tests were used to compare two continuous variables with a normal distribution. The Wilcoxon rank–sum test was used to compare two continuous variables with a non-parametric distribution. A *p*-value of <0.05 was considered significant for all tests. All statistical analyses were performed using STATA 18.0 (StataCorp. 2019. *Stata Statistical Software: Release 18*. College Station, TX: StataCorp LLC).

## 3 Results

Measures including VEU, absolute VEU, LVDI, and BIVAT-90 of each pacing configuration in all three settings (intact left bundle conduction, LBBB, and septal scar affecting the HPS) are summarised in Table 1.

**TABLE 1:** VEU, absolute VEU, LVDI, and BIVAT-90 values in response to each pacing configuration in three different settings (intact left bundle conduction, LBBB, and septal scar); (*) *p*-value comparing combined LBBP and combined LCPM configurations; and (**) *p*-value comparing RVOT-S vs. combined LBBP configurations.

	Baseline	PLBBP	DLBBP	RVOT-S	MS	AS	Combined LBBP	Combined LCPM	*p*-value*	*p*-value**
Intact left bundle conduction										
VEU	6.8 ± 4.6	−0.4 ± 4.5	−6.1 ± 4.1	21.7 ± 9.0	25.7 ± 7.5	25.2 ± 5.7	−3.3 ± 5.1	24.2 ± 7.6	<0.01	<0.01
Absolute VEU	6.9 ± 4.6	3.6 ± 2.5	6.3 ± 3.9	21.7 ± 9.0	25.7 ± 7.5	25.2 ± 5.7	5.0 ± 3.5	24.2 ± 7.6	<0.01	<0.01
LVDI	12.0 ± 1.6	12.0 ± 1.5	12.3 ± 1.6	22.1 ± 2.1	23.0 ± 2.9	24.1 ± 2.0	12.1 ± 1.6	23.0 ± 2.5	<0.01	<0.01
BIVAT-90	38.2 ± 4.3	40.9 ± 3.5	44.7 ± 3.0	70.1 ± 7.3	70.7 ± 9.4	74.9 ± 7.2	42.8 ± 3.8	71.9 ± 8.2	<0.01	<0.01
LBBB										
VEU	47.2 ± 5.6	−22.6 ± 3.2	−21.6 ± 3.3	21.7 ± 9.0	30.8 ± 6.0	25.2 ± 5.7	−22.1 ± 0.5	25.9 ± 7.9	<0.01	<0.01
Absolute VEU	47.2 ± 5.6	22.6 ± 3.2	21.6 ± 3.3	21.7 ± 9.0	30.8 ± 6.0	25.2 ± 5.7	22.1 ± 0.5	25.9 ± 7.9	<0.01	0.86
LVDI	24.4 ± 2.8	12.2 ± 1.6	12.4 ± 1.7	22.1 ± 0.1	23.1 ± 2.9	24.0 ± 2.0	12.3 ± 0.3	23.0 ± 0.3	<0.01	<0.01
BIVAT-90	78.9 ± 9.0	62.7 ± 5.6	62.0 ± 5.6	70.1 ± 7.3	70.7 ± 9.5	74.9 ± 7.2	62.3 ± 0.90	71.9 ± 1.1	<0.01	<0.01
Septal scar with non-conducting HPS										
VEU	44.1 ± 16.7	42.3 ± 19.5	41.0 ± 21.4	24.8 ± 9.0	35.7 ± 5.6	32.7 ± 6.4	41.7 ± 20.2	31.0 ± 8.4	<0.01	<0.01
Absolute VEU	44.1 ± 16.7	42.3 ± 19.5	41.0 ± 21.4	24.8 ± 9.0	35.7 ± 5.6	32.7 ± 6.4	41.7 ± 20.2	31.0 ± 8.4	<0.01	<0.01
LVDI	25.4 ± 6.6	25.4 ± 6.6	25.5 ± 6.5	24.5 ± 2.2	29.1 ± 2.4	27.9 ± 2.4	25.5 ± 6.5	27.2 ± 3.0	0.93	0.08
BIVAT-90	80.5 ± 22.1	80.2 ± 22.6	80.5 ± 22.2	75.7 ± 7.3	88.9 ± 7.9	87.5 ± 7.9	80.3 ± 22.1	84.0 ± 9.6	0.47	0.052

Abbreviations: LBBB, left bundle branch block; DLBBP, left bundle branch pacing at the level of left posterior fascicle; PLBBP, left bundle branch pacing at the level of the proximal left bundle; RVOT-S, right ventricular outflow tract-septal pacing; MS, mid-septal pacing; AS, apical-septal pacing; VEU, ventricular electrical uncoupling; LVDI, left ventricular dyssynchrony index; BIVAT-90, time taken to activate 90% of both ventricles.

### 3.1 Biventricular activation times

**FIGURE 2:**
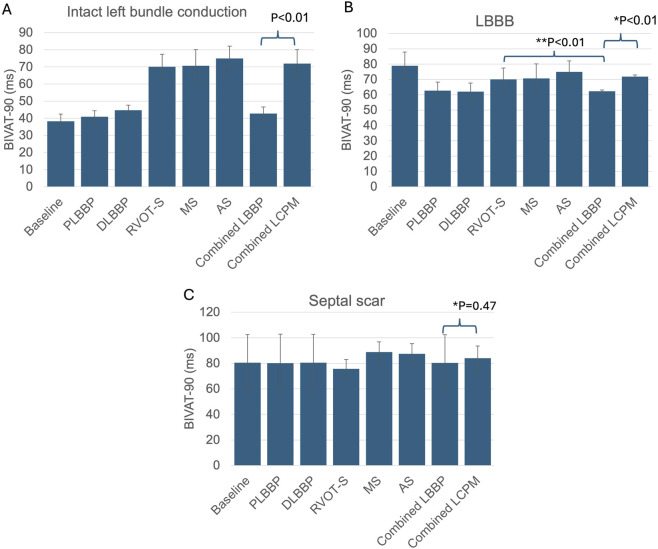
BIVAT-90 values in different settings: **(A)** intact left bundle conduction, **(B)** proximal left bundle branch block, and **(C)** septal scar affecting the HPS. (*) Comparison between combined LBBP and combined LCPM configurations; (**) comparison between RVOT-S and mean LBBP. PLBBP, proximal left bundle branch pacing; DLBBP, distal left bundle branch pacing; AS, leadless pacing at the apical septum level; MS, leadless pacing at the mid-septum level; RVOT-S, leadless pacing at the right ventricular outflow tract-septum level.

In the presence of normal left bundle conduction, combined LBBP configurations (PLBBP and DLBBP) produced lower BIVAT-90 than combined LCPM configurations (RVOT-S, MS, and AS) (42.8 ± 3.8 vs. 71.9 ± 8.2 ms, respectively; *p* < 0.01) (Figure 2A). In the presence of LBBB, combined LBBP configurations produced higher BIVAT-90 values but remained significantly lower than those of combined LCPM configurations (62.3 ± 0.9 vs. 71.9 ± 1.1 ms, respectively; *p* < 0.01) (Figure 2B). In the presence of a septal scar involving the HPS, there was no significant difference in BIVAT-90 between combined LBBP and combined LCPM configurations (80.3 ± 22.1 vs. 84.0 ± 9.6 ms, respectively; *p* = 0.47) (Figure 2C).

### 3.2 Interventricular dyssynchrony

**FIGURE 3:**
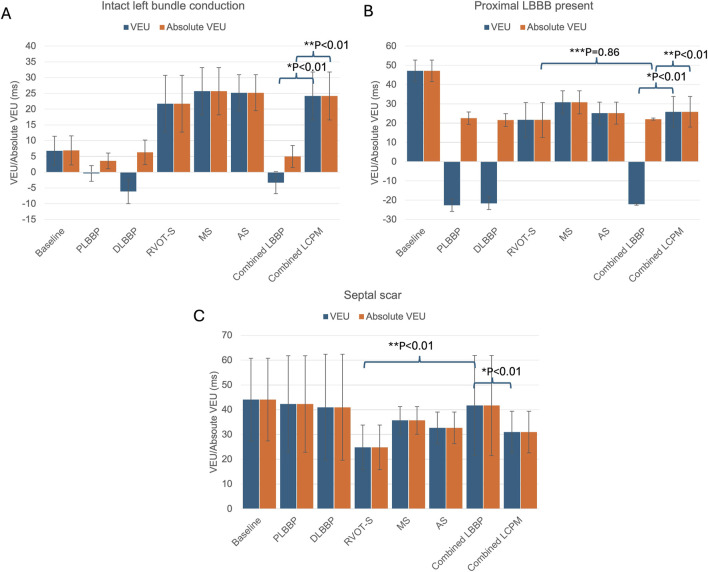
VEU values in different settings: **(A)** intact left bundle conduction, **(B)** proximal left bundle branch block, **(C)** and septal scar affecting the HPS. (*) Comparison of VEU between mean LBBP and mean LCPM, (**) comparison of absolute VEU between mean LBBP and mean LCPM, and (***) comparison of VEU between RVOT-S and mean LBBP. Blue charts denote VEU, and orange charts denote absolute VEU. PLBBP, proximal left bundle branch pacing; DLBBP, distal left bundle branch pacing; AS, leadless pacing at the apical septum level; MS, leadless pacing at the mid-septum level; RVOT-S, leadless pacing at the right ventricular outflow tract-septum level.

**FIGURE 4:**
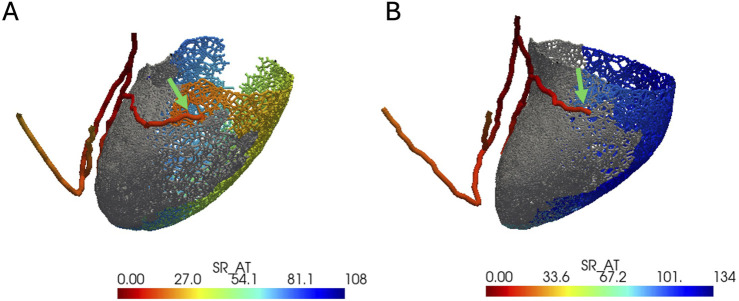
Anterior–posterior view of LV with an electrical activation map. Grey color denotes the area of a non-conducting scar. Red color denotes earliest activation, and dark blue denotes latest activation. The green arrow marks the terminal of the left anterior fascicle. **(A)** Heart model where the left anterior fascicle terminates beyond the area of the scar (where the Purkinje network is viable). **(B)** Heart model where the anterior fascicle terminates within the scar (where the Purkinje network is not viable).

In the presence of intact left bundle conduction, combined LBBP configurations had significantly lower VEU (−3.3 ± 5.1 vs. 24.2 ± 7.6 ms, respectively; *p* < 0.01) and absolute VEU (5.0 ± 3.5 vs. 24.2 ± 7.6 ms, respectively; *p* < 0.01) than those of combined LCPM configurations (Figure 3A). Similarly, in the presence of LBBB, combined LBBP configurations produced significantly lower absolute VEU (LBBP 22.1 ± 0.5 ms vs. LCPM 25.9 ± 7.9 ms; *p* < 0.01) than combined LCPM configurations (Figure 3B). However, when combined LBBP configurations were compared to RVOT-S alone, there was no significant difference in absolute VEU (RVOT-S 21.7 ± 9.0 vs. combined LBBP 22.1 ± 0.5 ms; *p* = 0.86). Conversely, in the setting of a non-conducting septal scar, combined LCPM configurations produced significantly lower VEU and absolute VEU than those of combined LBBP configurations (both VEU and absolute VEU: combined LCPM 31.0 ± 8.4 vs. combined LBBP 41.7 ± 20.2 ms; *p* < 0.01) (Figure 3C). Notably, in 6 of the 19 heart models, a proportion of Purkinje fibres supplied by the left anterior fascicle remained conductive as they were located beyond the area of scar tissue (Figure 4A). In these models, LBBP produced significantly lower VEU than the RVOT-S (combined LBBP 13.3 ± 4.9 vs. combined LCPM 31.0 ± 8.4 ms; P < 0.01). In the remaining 13 out of 19 heart models, both the anterior and posterior fascicles were within the non-conducting scar zone, with no activation of the Purkinje network (Figure 4B). This resulted in combined LCPM configurations producing less interventricular dyssynchrony than LBBP configurations (combined LCPM 31.3 ± 8.4 vs. combined LBBP 54.7 ± 5.4 ms; *p* < 0.01).

### 3.3 Intraventricular dyssynchrony

**FIGURE 5:**
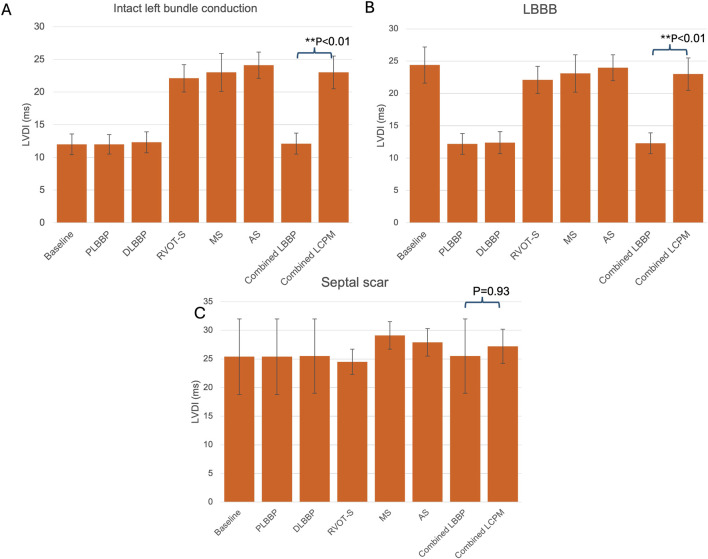
LVDI values in different settings: **(A)** intact left bundle conduction, **(B)** proximal left bundle branch block, and **(C)** septal scar involving the HPS. PLBBP, proximal left bundle branch pacing; DLBBP, distal left bundle branch pacing; AS, leadless pacing at the apical septum level; MS, leadless pacing at the mid-septum level; RVOT-S, leadless pacing at the right ventricular outflow tract-septum level.

In the presence of intact left bundle conduction, combined LBBP configurations produced significantly lower LVDI than that of combined LCPM configurations (12.1 ± 1.6 vs. 23.0 ± 2.5 ms, respectively; *p* < 0.01) (Figure 5A). Similarly, in the context of LBBB, LBBP configurations had significantly lower LVDI than LCPM configurations (12.3 ± 1.6 vs. 23.0 ± 2.5 ms, respectively; *p* < 0.01, Figure 5B). However, in the setting of a septal scar, combined LBBP configurations produced similar LVDI to combined LCPM configurations (25.5 ± 6.5 vs. 27.2 ± 3.0 ms, respectively; *p* = 0.93) (Figure 5C).

## 4 Discussion

### 4.1 Intraventricular dyssynchrony

LBBP produced improved intraventricular synchrony in the presence and absence of LBBB, but this effect on intraventricular synchrony was attenuated in the setting of a non-conducting septal scar. In the presence of a septal scar affecting the HPS, rapid conduction *via* the specialised cells of the Purkinje network is no longer feasible, resulting in slower conduction through the ventricular myocardium, which has a considerably lower inherent conduction velocity, and, consequently, markedly heterogeneous and prolonged LV activation times. This is consistent with the *in vivo* findings of Elliott et al. (2023), who reported that the presence of a septal scar is associated with an attenuation of LBBP’s beneficial effects on intraventricular dyssynchrony. In contrast, the effects of LBBP were not attenuated in the presence of proximal LBBB. Because the LBBB is located more proximally relative to the site of left bundle pacing, normal rapid activation of the left ventricle distal to the block is still possible *via* the specialised cells of the left bundle branches, resulting in significantly less heterogeneity in LV activation times.

### 4.2 Interventricular dyssynchrony

We computed both VEU and absolute VEU in our in silico modelling. VEU is a measure of interventricular dyssynchrony with directionality. A positive VEU indicates that the LV epicardial activation time is longer than the RV epicardial activation time, resembling LBBB, whereas a negative VEU indicates that the RV epicardial activation time is longer than the LV epicardial activation time, resembling RBBB. Absolute VEU simply reflects the degree of dyssynchrony between ventricles, without directionality.

Our *in silico* modelling demonstrated that combined LBBP configurations resulted in significantly less interventricular dyssynchrony (as reflected by VEU and absolute VEU) than combined LCPM configurations in the presence of intact left bundle branch conduction. In contrast, when proximal LBBB was present, combined LBBP configurations produced a greater amount of dyssynchrony, owing to later RV activation, than when left bundle branch conduction was intact. This prolonged RV activation is because, in the presence of proximal LBBB, activation of the right bundle *via* the left bundle is not possible during LBBP, and electrical propagation takes place *via* non-specialised cell-to-cell conduction, leading to delayed RV activation. This is reflected in the increase in the mean biventricular activation time (BIVAT-90) from 42.8 ms, in the presence of intact left bundle conduction, to 62.3 ms in the presence of proximal LBBB. Our *in silico* modelling finding replicates the *in vivo* finding of Ali et al. (2023a), who investigated the electrical response, using ECGi, of patients with LBBB to LBBP, His bundle pacing, and conventional biventricular pacing. They found that LBBP resulted in prolonged RV activation and, consequently, greater interventricular dyssynchrony than His bundle pacing, in which both the right and left bundles could be activated simultaneously. The prolonged RV activation in the presence of proximal LBBB also explains why combined LBBP configurations yielded similar absolute VEU values as LCPMs in the RVOT-S configuration–because both LBBP and RVOT-S produced a small amount of dyssynchrony but in opposite directions (VEU: combined LBBP: −22.1 ± 0.5 ms, RVOT-S: 21.7 ± 9.0 ms). This is important because, although much of the focus has been on pacing-induced left ventricular delay, pacing-induced right ventricular delay has also been linked to poor prognosis, including a higher risk of impaired haemodynamics and increased mortality (Hesse et al., 2001; Ploux et al., 2015; Sillanmäki et al., 2020). Although this issue may be mitigated with RV anodal capture, this requires significantly higher pacing output, resulting in impaired battery longevity with no significant improvement in biventricular haemodynamics (Ali et al., 2023b). In our simulations, we did not consider different types of pacing configurations (bipolar vs unipolar or RV anodal capture). Although RV anodal capture might provide better interventricular synchrony in some cases, it is not always clinically possible as it relies on direct contact of the anode electrode with the RV septum (Ali et al., 2023b). Therefore, we did not include this scenario in our study. Notwithstanding, Lu et al. (2023) found that implanting factors such as deployment of the lead tip in an oblique fashion and in the anterior-middle septum area increase the chances of successful RV anodal capture.

In the setting of a septal scar, LBBP configurations still resulted in a significant reduction in interventricular dyssynchrony in the models where the left anterior fascicle terminates beyond the area of scar tissue (6 out of 19). In the remaining 13 models where the left anterior fascicle terminated within scar tissue, the positive effect of LBBP on interventricular dyssynchrony is attenuated, and in these cases, LCPM configurations were superior in reducing interventricular dyssynchrony. This shows that the extent of a septal scar matters when implanting LBBP.

**FIGURE 6:**
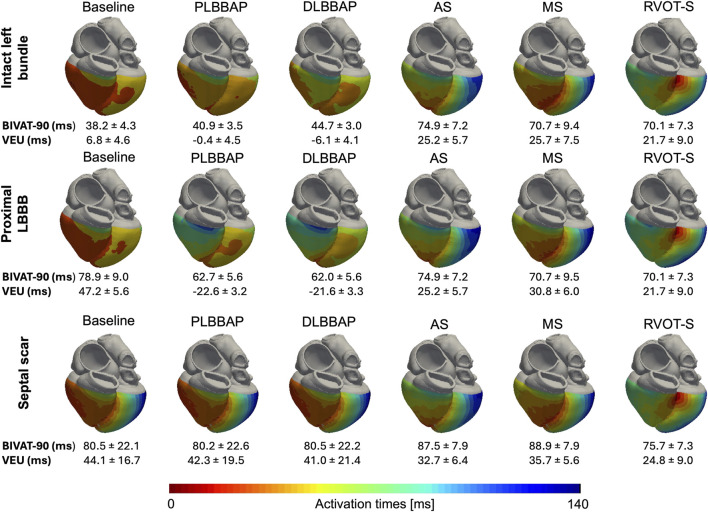
Anterior–posterior epicardial activation maps of each pacing configuration in different settings of one of the heart models. Top row: intact left bundle conduction. Middle row: presence of proximal LBBB at the level of the His bundle. RV activation is delayed during LBBP in the presence of proximal LBBB compared to that during intact left bundle conduction. Bottom row: septal scar rendering HPS non-conductive. PLBBP, LBBP at the level of proximal left bundle; DLBBP, LBBP at the distal left bundle; AS, leadless pacing at the apical septum level; MS, Leadless pacing at the mid-septum level; RVOT-S, Leadless pacing at the right ventricular outflow tract-septum level. Activation scale: the extreme left of the scale (dark red) denotes earliest activation (0 ms), and the extreme right of the scale (dark blue) denotes latest activation (140 ms). BIVAT-90 and VEU values are presented as the mean ± standard deviation.

Figure 6 illustrates the activation patterns in response to different pacing configurations in one of the heart models.

### 4.3 Septal scar involving the HPS

In the presence of a septal scar rendering the HPS nonconductive, LBBP resulted in more interventricular dyssynchrony than LCPM (VEU 41.7 ± 20.2 vs. 31.0 ± 8.4 ms, respectively, *p* < 0.01) with the RVOT-S configuration producing the lowest VEU (24.8 ± 9.0 ms) compared to all other LBBP and LCPM configurations. This is because in the majority (13 out of 19) of our heart models, where the scar renders the Purkinje fibres of the left bundle non-conducting, the activation wavefront travels retrogradely from the pacing stimulus up the left bundle into the right bundle, with RV depolarisation first, followed by LV depolarisation *via* non-specialised myocardial conduction. In contrast, pacing at the site of the RVOT-S, away from the scar tissue, affords a degree of biventricular activation *via* non-HPS septal myocardium, leading to overall lower interventricular dyssynchrony. In real-world cases, complete interruption of the HPS is relatively uncommon, and septal scars are often heterogeneous in their transmurality and conductivity. More than one location on the septum can usually be explored to achieve left bundle branch area capture when the initial deployment fails to achieve this. The findings from 6 out of 19 of our heart models, where a portion of the Purkinje network supplied by the left anterior fascicle located just beyond the area of scar remained conducting, demonstrate that LBBP may still have a beneficial effect in the presence of a less extensive septal scar. In rare cases where extensive transmural septal scarring occurs and renders the HPS non-conducting, such as in severe cases of septal viral myocarditis, advanced infiltrative diseases such as amyloidosis and sarcoidosis, and extensive myocardial infarction caused by proximal left anterior descending artery occlusion (Imran et al., 2010; Cheng et al., 2024), our modelling suggests that LCPM may yield less interventricular dyssynchrony than LBBP.

Interestingly, Elliot et al. investigated the effects of LBBP with the use of ECG in 10 patients (five had LBBB, one had RBBB, and four had RV-paced rhythm) and found that the presence of septal scar, either midwall or subendocardial, attenuated the resynchronisation effects of LBBP on the LV (i.e., reduced intraventricular synchrony). This suggests that the resynchronisation effects of LBBP may be reduced even when the septal scar is not transmural and the conductivity of the HPS is not completely abolished. Although Elliot et al. did not compare biventricular activation times or VEU between those with and without the septal scar, our *in silico* modelling suggests that LBBP effects on interventricular synchrony are also reduced by septal scarring.

### 4.4 Clinical implications

The implications of our *in silico* modelling are as follows:

1. LCPM is superior to LBBP with respect to interventricular synchrony in the presence of extensive septal scarring affecting the HPS. This finding is consistent with previous *in silico* and *in vivo* studies demonstrating an attenuation of the beneficial effects of LBBP on LV resynchronisation and positive remodelling in the presence of a septal scar (Chen et al., 2023; Elliott et al., 2023; Strocchi et al., 2023). This may make LCPM a more suitable option than LBBP in those with a ventricular pacing indication and preserved LV function, considering the absence of pocket- and lead-related complications and the increased technical complexity of LBBP implantation in the setting of septal scarring (Ponnusamy et al., 2020). Furthermore, the recent introduction of the Abbott AVEIR dual-chamber leadless pacemaker—which maintains atrioventricular (AV) synchrony—has positioned LCPM as a viable alternative for patients with persistent high-degree AV block (Knops et al., 2023). Although LCPM is associated with a lower overall risk of TR progression, studies have shown that implantation close to the tricuspid valve, such as in the high septal position, may increase the risk of TR progression (Salaun et al., 2018; Beurskens et al., 2019; Hai et al., 2021; Garweg et al., 2023; El-Chami et al., 2024; Yuyun et al., 2024). Therefore, further clarification is required through *in vivo* studies to determine whether the benefits of LCPM implantation in the RVOT-S position to minimise interventricular dyssynchrony are offset by the increased risk of TR progression.
2. There is possible equipoise between LBBP and RVOT-S in interventricular dyssynchrony in the presence of LBBB. First, LCPM at the RVOT-S position yielded similar absolute VEU values as LBBP. Second, even considering VEU (with directionality) instead of absolute VEU (without directionality), both LBBP and RVOT-S produced VEU values of <40 ms (combined LBBP −22.1 ± 3.3 vs. RVOT-S +21.7 ± 9.0 ms), below the threshold for the widely accepted definition for interventricular mechanical delay (Cleland et al., 2005). This is a new finding and warrants further *in vivo* comparison between LBBP and LCPM, specifically in the RVOT-S configuration, in patients with LBBB.

### 4.5 Limitations

The heart geometries used in this study were derived from healthy subjects to closely reflect the cardiac morphology of patients with preserved LV function and high-degree AV block. Therefore, results from this study may not be translatable to patients with heart failure with reduced ejection fraction. We did not simulate RV anodal capture in our *in silico* modelling. Although this may have mitigated delayed RV activation caused by LBBP in our simulation, particularly in the context of LBBB, real-world data suggest that it is not always clinically possible, comes at a considerable cost of significantly higher pacing output, and does not improve acute haemodynamics (Ali et al., 2023b). Similarly, although the use of epicardial biventricular pacing may reduce RV activation delay by optimising LV-RV delay in the context of LBBB, the aim of our study was to compare LCPM and LBBP in those with preserved LV function and complete AV block, where the use of conventional epicardial biventricular pacing is not guideline-recommended. In our study, the location of LBBB was simulated to be within the bundle of His. Therefore, the results of this modelling may not be translatable to scenarios where the location of LBBB is different, such as distal and diffuse LBBB. Depending on the level and nature of the block (focal or diffuse), left bundle capture or retrograde RV activation *via* the right bundle may or may not be possible, affecting VEU and overall biventricular activation times. In our modelling of proximal left bundle branch block, where the level of block is at the left intra-Hisian level, the activation wavefront starts in the RV and spreads across the septum slowly. If the activation wavefront came into contact with the LV Purkinje system, it was activated, and depolarisation within the LV could take place *via* the Purkinje network. It is unclear whether such Purkinje network activation takes place within the LV, but the close correlation of our generated *in silico* measurements of total ventricular activation time (TAT) with an *in vivo* study by Ploux et al. (2015) supports the validity of our left bundle branch block simulations.

Animal studies have shown that some Purkinje cells may survive an infarct with partial to complete recovery of function (Friedman et al., 1973; Garcia-Bustos et al., 2019; Sayers et al., 2025). In our models, Purkinje fibres that overlap the scar zone were treated as non-conducting under the assumption that Purkinje fibres are affected by hypoxia the same way as normal myocardium, to illustrate the impact of the most severe cases of myocardial scarring on the Purkinje system. Our *in silico* modelling results demonstrated that interventricular dyssynchrony can be minimised when LCPM is placed in the RVOT-S position. Although the high septal placement simulated in our *in silico* modelling is feasible (Garweg et al., 2023; Shantha et al., 2023; El-Chami et al., 2024), specific target deployment of LCPM onto the septum may not always be possible, particularly in smaller hearts. Our study did not account for the mechanical effects of lead implantation, particularly its impact on tricuspid valve function. Mechanical simulations are computationally more demanding, and their application remains limited to studies involving a small number of simulations. In the future, this study could be extended to include mechanics and investigate the effects of leadless pacing on valve function. Finally, this is a computational modelling study with a small number of heart models. Although statistical significance is presented, due to the small sample size, these values should be interpreted with caution.

## 5 Conclusion

To date, no direct comparison between LBBP and LCPM has been performed to investigate their effects on intraventricular and interventricular dyssynchrony, which are implicated in the development of pacing-induced cardiomyopathy. Our *in silico* modelling suggests that, in the presence of an extensive septal scarring rendering the Purkinje network non-conducting, LCPM is superior to LBBP in terms of interventricular synchrony, consistent with findings from previous studies. More interestingly, in the setting of LBBB, LCPM at a high septal position may be non-inferior to LBBP in interventricular dyssynchrony. Further *in vivo* studies are required to validate these findings.

## Data Availability

The original contributions presented in the study are included in the article/Supplementary Material; further inquiries can be directed to the corresponding author.
